# Prevalence of MRI findings in the cervical spine in patients with persistent neck pain based on quantification of narrative MRI reports

**DOI:** 10.1186/s12998-019-0233-3

**Published:** 2019-03-06

**Authors:** Rikke Krüger Jensen, Tue Secher Jensen, Søren Grøn, Erik Frafjord, Uffe Bundgaard, Anders Lynge Damsgaard, Jeppe Mølgaard Mathiasen, Per Kjaer

**Affiliations:** 1 Nordic Institute of Chiropractic and Clinical Biomechanics, Odense, Denmark; 2 Medical Department, Spine Centre of Southern Denmark, Lillebaelt Hospital, Middelfart, Denmark; 3 Department of Sports Science and Clinical Biomechanics, University of Southern Denmark, Odense, Denmark; 4 Department of Diagnostic Imaging, Silkeborg Regional Hospital, Silkeborg, Denmark; 5 Department of Clinical Medicine, Aarhus University, Aarhus, Denmark; 6 Department of Applied Health Research, University College Lillebaelt, Odense, Denmark

**Keywords:** MRI, Cervical spine, Prevalence

## Abstract

**Background:**

Previous studies of patients with neck pain have reported a high variability in prevalence of MRI findings of disc degeneration, disc herniation etc. This is most likely due to small and heterogenous study populations. Reasons for only including small study samples could be the high cost and time-consuming procedures of having radiologists coding the MRIs. Other methods for extracting reliable imaging data should therefore be explored.

The objectives of this study were 1) to examine inter-rater reliability among a group of chiropractic master students in extracting information about cervical MRI-findings from radiologists´ narrative reports, and 2) to describe the prevalence of MRI findings in the cervical spine among different age groups in patients above age 18 with neck pain.

**Method:**

Adult patients with neck pain (with or without arm pain) seen in a public hospital department between 2011 and 2014 who had an MRI of the cervical spine were identified in the patient registry ‘SpineData’. MRI-findings were extracted and quantified from radiologists’ narrative reports by second-year chiropractic master students based on a set of coding rules for the process.

The inter-rater reliability was quantified with Kappa statistics and the prevalence of the MRI findings were calculated.

**Results:**

In total, narrative MRI reports from 611 patients were included. The patients had a mean age of 52 years (SD 13; range 19–87) and 63% were women. The inter-observer agreement in coding MRI findings ranged from substantial (κ = 0.78, CI: 0.33–1.00) to almost perfect (κ = 0.98, CI: 0.95–1.00).

The most prevalent MRI findings were foraminal stenosis (77%), uncovertebral arthrosis (74%) and disc degeneration (67%) while the least prevalent findings were nerve root compromise (2%) and Modic changes type 2 (6%). Modic type 1 was mentioned in 25% of the radiologists’ reports. The prevalence of all findings increased with age, except disc herniation which was most prevalent for patients in their forties.

**Conclusion:**

MRI-findings from radiologists’ narrative reports can reliably be extracted by chiropractic master students with a minimum of training. Degenerative findings in the cervical spine were most commonly found at levels C5/C6 and C6/C7 and increased with age.

**Electronic supplementary material:**

The online version of this article (10.1186/s12998-019-0233-3) contains supplementary material, which is available to authorized users.

## Background

Globally, neck pain is ranked as the fourth cause of years lived with disability in reports from both 1990 and 2013 [1]. In 2013, neck pain was the second leading cause of years lived with disability in Denmark, only exceeded by low back pain [1]. Neck pain is a very common condition with an estimated mean one year prevalence of 25% [2].

The use of Magnetic Resonance Imaging (MRI) in the search for biological causes of neck pain remains controversial as studies have shown that pathoanatomical changes in the cervical spine are also common in healthy volunteers [3], even though certain MRI findings appear more prevalent in people with pain compared to people without pain [4–7]. In addition, there is no available evidence supporting MRI findings as predictive for treatment effect in people with neck pain, although one study has reported that neck pain related activity limitations could be related to MRI findings [8].

There is a wide variability in the reported prevalence of different MRI findings such as for example disc degeneration [7, 9–11] and Modic changes [4–7] and only limited knowledge is available to inform the clinician about what should be expected of MRI findings at a certain age and how this relates to neck pain in different populations. There are several potential reasons for the variability in the prevalence of MRI findings such as differences in patient populations and age groups, sampling methods, as well as rating criteria for the images. Using a large sample of patients from the same treatment setting who have a wide age span to cover age related degenerative findings will enable us to establishing consistent prevalence rates of a broad range of MRI findings in the cervical spine.

The use of quantitative coding of images is a way to ensure high quality and reliability of the MRI data and is the preferred method applied in research projects [12–15]. However, using a direct coding of image finding is not always applicable because it is time consuming and there are only few research radiologists.

As an alternative to this procedure, Kent et al. [16] investigated if physiotherapy students could extract and quantify MRI data from narrative radiologists’ reports of the lumbar spine and found the method valid and with high reliability. However, it remains uncertain how this method will perform in other groups of students and in other anatomical areas.

For these reasons, the objectives of this study were to 1) to examine inter-rater reliability among a group of chiropractic master students in extracting and quantifying information from radiologist-generated MRI narrative reports, and 2) to describe the prevalence of MRI findings in the cervical spine in different age groups of patients above age 18 with neck pain.

## Method

### Study design

This study was a retrospective cross-sectional observational study.

### Patient population

All participants were above the age of 18 and had neck pain with or without arm pain. They had all attended the same publicly funded outpatient spine clinic (The Spine Centre of Southern Denmark) where they had been referred to from the primary care chiropractors and general practitioners for a multidisciplinary evaluation.

### Data collection

**Fig. 1:**
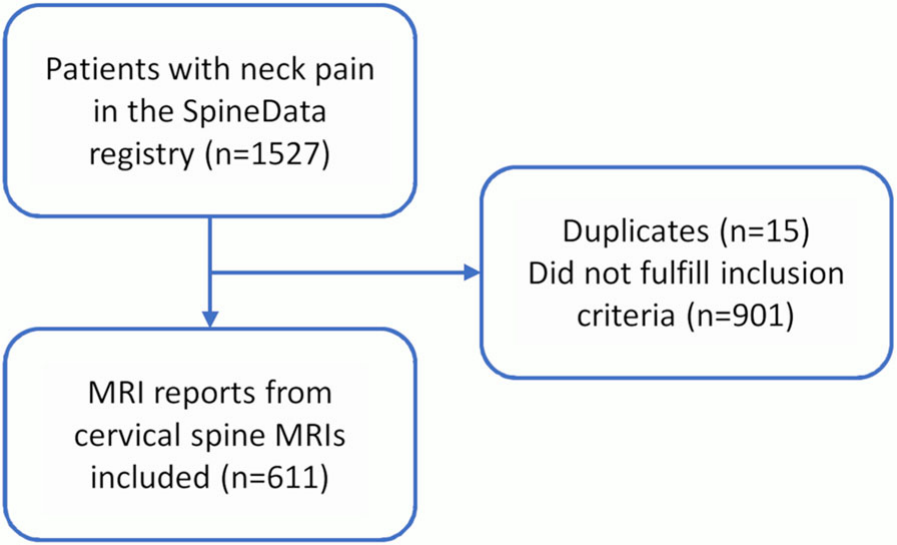
Flowchart of the selection and inclusion process of individual patients’ MRI data

Clinical data are routinely collected at the spine clinic using an electronically based standardised questionnaire ‘SpineData’ which is an internet-based, multiuser registry designed to capture patient data electronically at the point of clinical contact [17]. A list of patients > 18 years with neck and with or without arm pain, who had attended the spine clinic between January 1, 2011 and December 31, 2014, was extracted from the SpineData registry and the listed patients were assessed for eligibility. Patients who had filled out the questionnaire at the first visit and at 12-months follow-up, and also had an MRI of the cervical spine from the radiology department of the hospitals in either Middelfart or Vejle, were included. The narrative radiologists’ reports were collected and evaluated. Patients were excluded if one or more of the following pathoanatomies were identified: recent acute vertebral fractures, surgical fusions, spinal infections, tumors, inflammatory spondyloarthropathy or other serious pathology. A summary of the data collection is presented in Fig. 1.

### Imaging

Only MRIs from the hospital’s radiology departments of Middelfart and Vejle were included in order to ensure homogeneity in the MRI scan and MRI evaluation protocols. The MRI protocol included the following sequences: one localiser, a sagittal short-tau inversion recovery (STIR), a sagittal T1-weighted turbo spin-echo (TSE), a sagittal T2-weighted Volume Isotropic Turbo spin echo Acquisition (VISTA) and axial T2 weighted TSE of the three lower cervical segmental levels with supplement of segmental levels suspicious of pathology. In addition, the radiologists use reconstructed semi-coronal series from the VISTA sequence, as part of their standardised evaluation, for visualisation of the neural foramina. The majority of MRIs were performed with a 1.0 Tesla (Philips Panorama, Best, The Netherlands) or 1.5 Tesla unit (Philips Achieva and Philips Ingenia, Best, The Netherlands) MRI system or, although rarely, with a 0.2 Tesla unit.

The narrative reports were retrieved from this hospitals Picture Archiving and Communication System (PACS) EasyViz archive (Medical Insight, Valby, Denmark).

### Variables of interest

Ten MRI findings were chosen for this study as they were considered relatively common and relevant to the research context of this study; Modic changes type 1 and type 2, disc degeneration, disc herniation, disc bulge, nerve root compromise, foraminal stenosis, central stenosis, facet joint arthrosis and uncovertebral arthrosis.

Descriptive clinical data on age, sex, sick leave, neck and arm pain as measured on an 11-point numeric rating scale [18] and physical function measured with the Neck Disability Index (NDI) [19] were available from the SpineData registry.

### Coding of MRI narrative reports

Coding of the narrative reports was modified for the cervical spine from the process previously described by Kent et al. [13]. A set of coding rules was developed to record the presence or absence of an MRI finding on each of the cervical segmental levels from C2/C3 to C7/Th1. A segmental level was defined as the inferior endplate of the top vertebra to the superior endplate of the vertebra below including the inter-vertebral space (e.g. inferior endplate of C2 to the superior endplate of C3).

The reviewers of the narrative reports were six second-year chiropractic master students who had finished their imaging course of 16 ECTS points (European Credit Transfer and Accumulation System). The data collection was part of their pre-graduate Master Thesis project which they had chosen from a catalogue of research projects suitable for a Master Thesis. The subject was chosen out of availability and interest. The students described the coding rules for identifying each of the MRI findings based on i) previous work in the department [16], ii) a consensus document from the radiological department and iii) by consulting senior researchers. In-person meetings were conducted first between the groups of students and later with the attendance of a senior researcher to review and refine the final set of coding rules. An additional file shows the final set of coding rules (see Additional file 1).

### Reliability testing

The reliability study was done in two steps. One study was performed to test the inter-rater reliability between four of the six chiropractic master students for data extraction of disc degeneration, disc herniation, disc bulge, nerve root compromise, foraminal stenosis, central stenosis, facet joint arthrosis and uncovertebral arthrosis. The study was based on six cervical segmental levels in 59 patients (354 levels). Another study investigated the reliability of the data extraction of Modic changes type 1 and type 2 on six levels in 50 patients (300 segmental levels) between the remaining two students. Each finding from the MRI radiologists’ reports was registered in spread sheets developed for this purpose (Microsoft Excel 2010, Microsoft Corp, Redmond, WA, USA). To ensure blinding the observers were allocated to different rooms and filled in the spread sheets independently. Each observer then delivered the data to one of the senior researchers who merged the data and performed the analysis.

### Statistics

Unweighted Kappa statistics including 95% confidence intervals (CI) were used to quantify the inter-rater reliability. The interpretation of the Kappa coefficient (κ) was based on Landis and Koch for strength of agreement: values lower than 0.20 indicated slight agreement; 0.21–0.40, fair agreement; 0.41–0.60 moderate agreement; 0.61–0.80 substantial agreement; and 0.81–1.00 almost perfect agreement [20]. The prevalence of the MRI findings for each segmental level and per person was calculated in percentage. The age of the participants was divided into decades. The minimum and maximum age group were merged with nearest age group if < 10 per group. STATA 14 (Stata Corp, College Station, Texas, USA) was used for all data management. The prevalence of MRI findings was calculated by disc level (C2/C3 to C7/Th1), by patient level and by age (18–30, 31–40, 41–50, 51–60, 61–70 and 70+ years of age).

## Results

A total of 1527 patients with neck pain and baseline questionnaire were identified in the SpineData registry within the predefined time period. After excluding duplicate patient episodes, patients without MRI and patients without follow-up data, 611 narrative MRI reports were included in the final sample. Of these, none had severe pathology on MRI. Figure 1 illustrates the inclusion of patients.

The patients had a mean age of 52 years (SD 13; range 19–87) and 63% were women. Baseline demographics and clinical characteristics are shown in Table 1.

**Table 1:** Descriptive data of the study sample

Patient characteristic (*n* = 611)	
Age (years), mean (SD)	52 (13)
Age categories (years), n	
19–30	33
31–40	85
41–50	135
51–60	187
61–70	123
71–87	48
Women (%)	63
Sick leave within 3 months (%)	40
Neck pain intensity, (NRS 0–10), mean (SD)	5.5 (2.5)
Arm pain intensity, (NRS 0–10), mean (SD)	4.5 (3.1)
Activity limitation (NDI) (%), mean (SD)	38 (17)

*NRS* Numerical rating scale, *NDI* Neck Disability Index, *SD* Standard Deviation

The inter-observer agreement ranged from substantial (κ = 0.78, 95% CI 0.33–1.0) to almost perfect (κ = 0.98, CI 0.95–1.00). For nine out of ten of the MRI variables the kappa values were above 0.9. Only ‘nerve root compromise’ was below 0.9 with κ = 0.78 (95% CI 0.33–1.0). Kappa values with 95% confidence intervals for each of the variables are shown in Table 2.

**Table 2:** Inter-rater reliability in coding MRI findings from narrative reports

MRI finding	Kappa coefficient	95% CI
Modic changes type 1	0.97	0.92–1.00
Modic changes type 2	0.96	0.87–1.00
Disc degeneration	0.96	0.93–0.98
Disc herniation	0.91	0.80–0.98
Disc bulge	0.94	0.90–0.97
Nerve root compromise	0.78	0.33–1.00
Foraminal stenosis	0.97	0.95–0.99
Central stenosis	0.94	0.88–0.97
Facet joint arthrosis	0.98	0.95–1.00
Uncovertebral arthrosis	0.96	0.93–0.98

Inter-rater reliability was estimated with two readers for Modic changes type 1 and type 2 (six segmental levels from 50 patients) and with four readers for the rest of the variables (six segmental levels from 59 patients).

The most prevalent reported finding was foraminal stenosis (77%), uncovertebral arthrosis (74%) and disc degeneration (67%) while the least prevalent finding was nerve root compromise (2%) and Modic changes type 2 (6%). Modic changes type 1 was mentioned in 25% of the reports. All variables had the highest prevalence at level C5/C6 and C6/C7 except from facet joint arthrosis which was mostly present at level C3/C4 and C4/C5. Thirteen percent of patients did not have any findings on the MRI and 73% had the presence of any MRI finding on more than one level. The prevalence of findings in total and per level is presented in Table 3.

**Table 3:** Prevalence of cervical MRI findings per segment and in total (n = 611)

MRI finding	C2/C3	C3/C4	C4/C5	C5/C6	C6/C7	C7/Th1	Total^a^	> 1 level
Modic type 1	1.5%	5.4%	6.4%	12.3%	12.3%	1.8%	25%	8%
Modic type 2	1.2%	1.5%	1.8%	3.4%	3.9%	1.3%	6%	2%
Disc degeneration	2.0%	10.1%	25.5%	57.9%	41.7%	3.3%	67%	45%
Disc herniation	0.0%	0.7%	2.6%	7.7%	6.4%	0.5%	16%	2%
Disc bulge	0.2%	7.5%	15.4%	36.7%	27.7%	0.7%	51%	27%
Nerve root compromise	0.0%	0.2%	0.3%	1.2%	0.7%	0.0%	2%	0%
Foraminal stenosis	6.4%	30.1%	39.3%	64.8%	50.1%	3.8%	77%	59%
Central stenosis	0.2%	3.1%	9.5%	28.5%	19.8%	0.3%	38%	18%
Facet joint arthrosis	9.5%	18.5%	18.8%	16.4%	10.3%	3.9%	37%	22%
Uncovertebral arthrosis	4.3%	29.8%	37.3%	62.5%	45.0%	2.6%	74%	56%

^a^Patients with MRI findings in one or more segmental levels

**Fig. 2:**
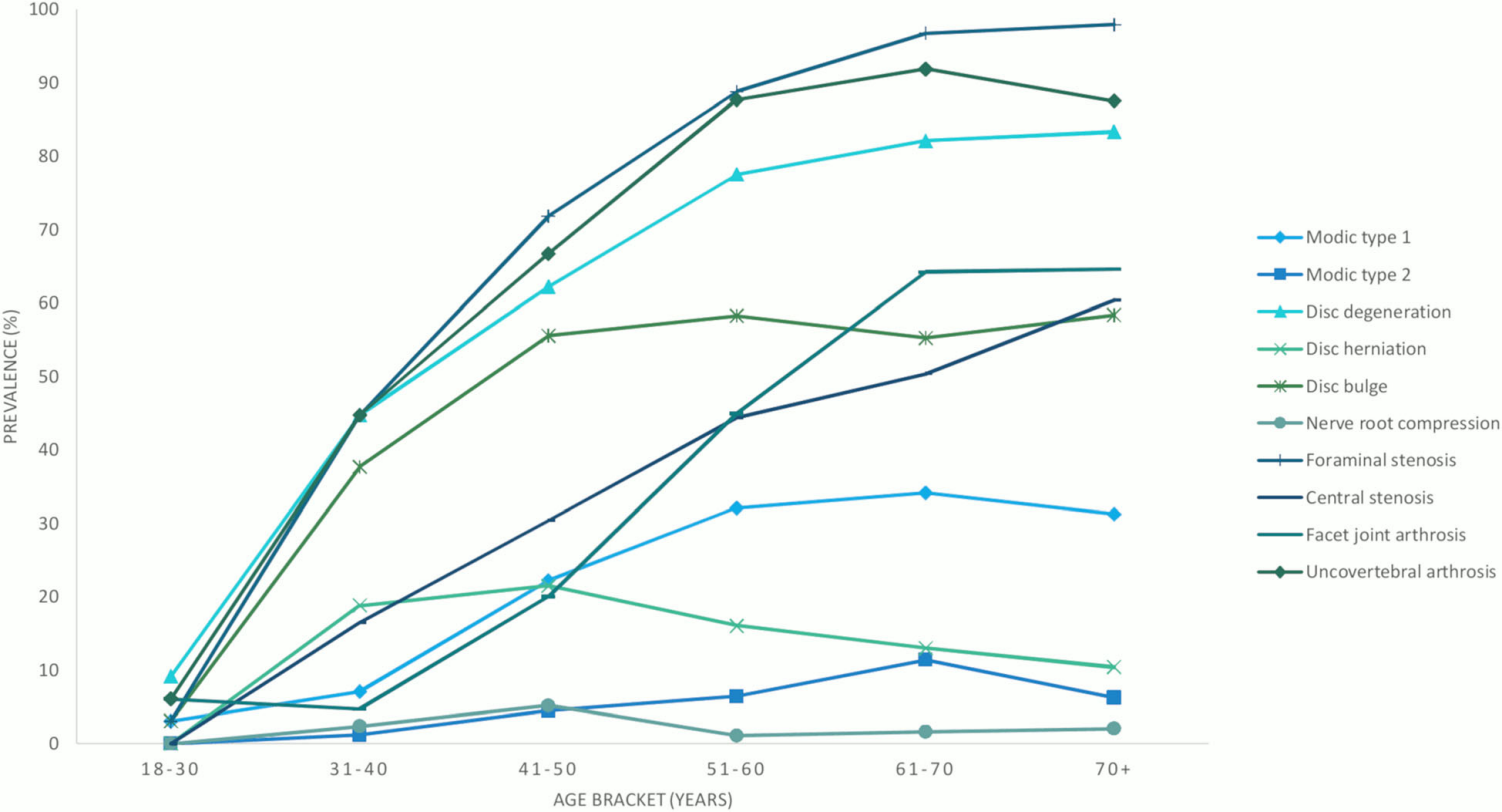
Prevalence of cervical MRI findings according to age categories in patients with neck pain

Figure 2 displays the prevalence of MRI findings by age categories. The figure indicates that MRI findings such as disc degeneration, disc bulge, uncovertebral arthrosis and foraminal stenosis starts to increase in the twenties while central stenosis, facet joint arthrosis and Modic changes type 1 increases for patients in their thirties and disc herniation is almost evenly distributed across the age groups with a small peak in patients in their forties.

## Discussion

To our knowledge this is the first study to apply this data extraction method from radiology reports of the cervical spine. The results show that chiropractic students at a master’s level can, with a minimum of training, extract reliable data from narrative reports, as the intra-observer agreement ranged from substantial to almost perfect. The prevalence study showed that degenerative MRI-findings are common in patients with neck pain. Three quarters of the patients had foraminal stenosis, uncovertebral arthrosis or disc degeneration, half of them had disc bulges, one quarter to one third had Modic changes type 1, facet joint arthrosis or central stenosis and very few (2–6%) had nerve root compromise or Modic changes type 2.

### Inter-rater reliability

Kent et al. [16] have previously developed and tested the method on data extraction from MRI reports of the lumbar spine and found similar results. In that study, three physiotherapy students evaluated four sets of 20 MRI reports across 14 pathoanatomical categories and found kappa values ranging from substantial to almost perfect agreement. As in the present study nerve root compromise was the only variable not exceeding an inter-rater kappa value above 0.9. This might be explained by the variety in the language used in the narrative reports to describe this finding. At the same time, nerve root compromise was found to be the least prevalent MRI finding (2%) in the cervical spine which gives the readers less training material.

The method of extracting data from narrative radiology reports has previously been investigated using computer-assisted categorisation of human language. Although the technique is quite advanced compared to students extracting the data it is based on the same recognition of predefined words or phrases. This area of research called natural language processing has proven to be useful in detecting the presence of a finding or disease from unstructured reports. In a systematic review Pons et al. [21] identified 67 publications describing information extraction and conclude that the performance of natural language processing applications in radiology is generally high.

### Prevalence

In a study by Karki et al. [10] with 750 patients with neck pain the reported prevalence of foraminal stenosis was only 41% compared to 77% in our population. For disc degeneration Karki et al. [10] reported the prevalence to be 76% versus 67% in our population which is comparable. The difference in prevalence of foraminal stenosis could be that the study by Karki et al. used a low field MRI system (0.35 T) and did not use semi-coronal sequences for the visualisation of the neural foramina. Also, there was a difference in mean age of the populations with our population being on average seven years older and as degenerative findings increases with age this could also influence the difference in prevalence.

The prevalence seems to be less in non-clinical populations as Matsumoto et al. [22] found the prevalence of foraminal stenosis to be present in only 5.9% of cervical disc levels in 497 asymptomatic volunteers and Arnbak et al. found a prevalence of disc degeneration in the cervical spine to be only 24% [5] in a population of 1037 patients between 18 and 40 years of age seeking care for low back pain. However, as the mean age of this study sample was 52 years the prevalence would be expected to be higher due to age alone.

Peterson et al. reported Modic changes type 1 in 16% and type 2 in 3.5% in patients with neck pain [6]. This is almost in line with our prevalence of 25% for type 1 and 6% for type 2. Another study by Mann et al. [7] investigating 500 patients over the age of 50 with neck pain also found a comparable prevalence of type 1 (15%) while type 2 was as high as 28%.

Modic changes also seem to be more prevalent in the cervical spine in people with neck pain (8.8%) when compared to people without (3.3%) [4]. Also, in the study by Arnbak et al. [5] the prevalence of Modic changes type 1 was reported to be only 2% and type 2 1.7%, however, as this was a young population and as the prevalence of Modic changes seems to increase with age, the numbers are not directly comparable. In a study by Bendix et al. [23] comparing low-field and high-field MRI machines when investigating Modic changes in the lumbar spine the authors found that type 1 was detected three times more often using a low field MRI while type 2 was detected two times more often when using high field MRI. In the present study the high field systems (1.0 Tesla and 1.5 Tesla) were primarily used and only rarely was the low field system (0.2 Tesla) used.

Although some MRI findings seem to be more prevalent in symptomatic people compared to those without neck pain, the limitations of the studies described above prevent a clear conclusion. Also, as this is a cross sectional study of a population with neck pain, the data cannot inform the association between MRI findings and the presence of symptoms, e.g. neck pain.

### Strengths and limitations

The strength of this study is the size of the study sample, which provides us with enough data to report on even less frequent findings such as nerve root compromise. Also, the high agreement found in the data extraction process showed that the reporting of the radiologist is sufficiently quantified in this dataset. However, we do not know the agreement between radiologists describing the original MRIs which is a major limitation of the quality of the data. On the other hand, this is the material on which clinician’s make decisions in everyday practice in the meeting with the patient. Also, the radiology departments have a consensus document describing the content of a radiologist report on an MRI recording of the spine (document in Danish is available on request). The consensus document describes the most common MRI pathologies such as disc degeneration, disc herniation, central and foraminal stenosis and are based on international classifications [24, 25]. For example, disc degeneration is evaluated as four categories: 1) ‘normal’ (normal disc height and disc signal intensity), 2) ‘mild disc degeneration’ (slightly reduced disc height and decreased disc signal intensity), 3) ‘moderate disc degeneration’ (moderate reduced disc height) and 4) ‘severe disc degeneration’ (collapsed disc space).

Although the present study has high internal validity, it is questionable to what extent the results from this study can be applied more generally as the variability in the interpretation of MRI scans is far from standardised. In a recent study [26] from the US, one 63-year old patient with low back pain had lumbar spine MRIs performed at ten different MRI centres over a period of 3 weeks. Comparison of the 10 narrative reports revealed considerable variability; none of the 49 described findings occurred in all 10 reports and only one finding occurred in nine reports. In order to use the method of extracting data from narrative reports, as described in the present study, one must make sure that the reporting is standardised.

### Perspectives

The method presented in this study of using inexperienced people to reliably extract data from narrative reports is a relatively simple way to collect large amounts of imaging data that can be used for research and quality assurance purposes. The prevalence estimates that we have reported for different segmental levels and age categories can provide clinicians and researchers with information on the expected prevalence for specific spinal pathoanatomical findings in patients with neck pain. Overdiagnosis may occur when a medical diagnosis is based on age-related MRI findings. It is therefore important for clinicians to know and communicate to the patient exactly how ‘normal’ these findings are in a patient population in order to avoid incorrect interpretation of MRI findings to be the cause of a patient’s symptoms. Further studies are needed to clarify the clinical relevance of these findings. Also, as spinal MRI findings obviously increase with age researchers should be conscious to report on prevalence according to age categories.

## Conclusion

Narrative radiologists’ reports can reliably be quantified by chiropractic master students with a minimum of training. Degenerative findings in the cervical spine were very common and mentioned in approximately 70% of the radiologists’ reports while nerve root compromise was a rare finding.

## Additional file

Additional file 1: The final set of decision rules used to extract pathoanatomic findings from MRI narrative reports. The file shows the final coding rules for identifying each of the MRI findings from the narrative reports. (PDF 118 kb)

## Abbreviations

CI: Confidence Interval
ECTS: European credit transfer and accumulation system
MRI: Magnetic resonance imaging
NDI: Neck disability index
NRS: Numerical rating scale
SD: Standard deviation
STIR: sagittal short-tau inversion recovery
TSE: turbo spin-echo
VISTA: Volume isotropic turbo spin echo acquisition
κ: Kappa coefficient
