# Supplementary material for: Prevalence of MRI findings in the cervical spine in patients with persistent neck pain based on quantification of narrative MRI reports
Source: Chiropr Man Therap. 2019 Mar 6;27:13. doi: 10.1186/s12998-019-0233-3 (PMC6402131; doi:10.1186/s12998-019-0233-3)
Supplement: Supplementary file 1 — The final set of decision rules used to extract pathoanatomic findings from MRI narrative reports. The file shows the final coding rules for identifying each of the MRI findings from the narrative reports. (PDF 118 kb) [file 12998_2019_233_MOESM1_ESM.pdf]

Additional file 1 - The final set of decision rules used to extract pathoanatomic findings from MRI narrative reports

Pathology	Expressions used in the narrative reports
**Modic changes type 1**	– Modic changes type 1<br>– Vertebral endplate signal change type 1<br>– Endplate changes type 1<br>– Increased signal intensity in the endplate
**Modic changes type 2**	– Modic changes type 2<br>– Vertebral endplate signal change type 2<br>– Endplate changes type 2<br>– Fatty degeneration in the endplate
**Disc degeneration**	– Light/moderate/severe degeneration<br>– Slight/moderate reduced disc height<br>– Collapsed disc space<br>– Reduced disc signal intensity<br>– Spondylosis<br>– Collapsed intervertebral space<br>– Age related degeneration
**Disc herniation**	– Herniation<br>– Extrusion<br>– Sequestration<br>– Prolapse
**Disc bulge**	– General/broad-based/focal bulge<br>– Protrusion<br>– Prolapse-like protrusion
**Nerve root compromise**	– Dislocation of nerve root<br>– Entrapment<br>– Impression<br>– Reduced space<br>– Compression<br>– Compromise<br>– Affected nerve root<br>➢ (NO nerve root compromise is registered if the following expressions are used: ‘touching’, ‘contact’, ‘enhancement’)
**Foraminal stenosis**	– Mild/moderate/severe/pronounced/absolute foraminal stenosis<br>– Reduced perineural fat signal<br>– No visible perineural fat<br>– Reduced foraminal area<br>– Formation of osteophytes in foramen
**Central stenosis**	– Mild/moderate/severe/pronounced/absolute central stenosis<br>– Reduced fluid around nerve roots or medulla<br>– No visible fluid around nerve roots or medulla

	– Reduced space in central canal<br>– Canal stenosis<br>– Spinal stenosis<br>– Myelopathy
**Facet joint arthrosis**	– Mild/moderate/severe arthrosis<br>– Osteophytes in relation to facet joint<br>– Narrowing of joint space<br>– Cysts in relation to facet joint<br>– Degenerative changes<br>– Age related facet joint changes<br>– Ankylosis<br>– Spondylarthrosis
**Uncovertebral arthrosis**	– Mild/moderate/severe arthrosis<br>– Osteophytes in relation to uncovertebral joint
**Exclusion criteria**	– Subluxation<br>– Cancer<br>– Infection<br>– Inflammatory arthritis<br>– Fracture<br>– Previously surgery in cervical spine<br>– Other serious pathology

*The original document is in Danish and has been translated for this publication
